# Cranial-Vertebral-Maxillary Morphological Integration in Down Syndrome

**DOI:** 10.3390/biology11040496

**Published:** 2022-03-24

**Authors:** Marta Teresa García-García, Pedro Diz-Dios, María Teresa Abeleira-Pazos, Jacobo Limeres-Posse, Eliane García-Mato, Iván Varela-Aneiros, Mercedes Outumuro-Rial, Márcio Diniz-Freitas

**Affiliations:** Medical-Surgical Dentistry Research Group (OMEQUI), Health Research Institute of Santiago de Compostela (IDIS), University of Santiago de Compostela (USC), 15782 Santiago de Compostela, Spain; mmgarcias@hotmail.com (M.T.G.-G.); pedro.diz@usc.es (P.D.-D.); maite.abeleira@usc.es (M.T.A.-P.); eliane.garma@gmail.com (E.G.-M.); ivan.varela.aneiros@gmail.com (I.V.-A.); mercedesoutumuro@hotmail.com (M.O.-R.)

**Keywords:** down syndrome, atlantoaxial joint, skull base, cephalometry, developmental instability, canalization

## Abstract

**Simple Summary:**

Phenotypic variability can be structured according to three interrelated components: developmental stability, morphological integration, and canalization. The cranium presents modular organization, consistent with the principles of morphological integration. In Down syndrome (DS), the most common genetic aneuploidy, the integration of the cranial-vertebral-maxillary complex, remains unknown. This study aimed to analyze whether there are significant relationships between the skull base, atlantoaxial joint, and maxillary-mandibular complex in a study group of 41 individuals with DS and nonsyndromic controls. Twenty-nine measurements were performed on each participant’s cone-beam computed tomography images, which were grouped into three blocks: atlantoaxial dimensions, craniovertebral dimensions, and cephalometric dimensions. With regard to the association between blocks, we found no significant relationship in the DS group. However, we confirmed a statistically significant correlation between all blocks of variables in the controls. In conclusion, these results confirm a very poor morphological integration of the cranial-cervical-maxillary complex in individuals with DS. This finding reinforces the proposal that gene overload enhances canalization, which could potentially affect the outcomes of certain orthopedic and surgical procedures.

**Abstract:**

Background: Morphological integration refers to the tendency of anatomical structures to show correlated variations because they develop in response to shared developmental processes or function in concert with other structures. The objective of this study was to determine the relationships between the dimensions of different cranial-cervical-facial structures in patients with Down syndrome (DS). Methodology: The study group consisted of 41 individuals with DS who had undergone cone-beam computed tomography (CBCT) at the Dental Radiology Unit of the University of Santiago de Compostela (Spain). In the historical archive of this same unit, 41 CBCTs belonging to individuals with no known systemic disorders or severe malformations of the maxillofacial region were selected, forming an age and sex-matched control group. Twenty-nine measurements were performed on each participant’s CBCT images, which were grouped into three blocks: atlantoaxial dimensions, craniovertebral dimensions and cephalometric dimensions. To determine whether there were significant differences between the dimensions obtained in the DS and control groups, we applied multiple analysis of variance and linear discriminant analysis tests. The analysis of the association between blocks (in pairs) was performed with the canonical correlation analysis test. Results: The dimensions evaluated in the three blocks of variables of individuals with DS differ significantly from those of nonsyndromic controls (*p* < 0.001). The highest discriminative capacity to identify controls and patients with DS was obtained with the cephalometric dimensions (87.5%). With regard to the association between blocks (two-by-two measurements), we found no significant relationship in the DS group. However, we confirmed a statistically significant correlation between all pairs of blocks of variables in the controls, especially between the atlantoaxial and cephalometric dimensions (*p* < 0.001) and between the craniovertebral and cephalometric dimensions (*p* < 0.001). Conclusions: Our results confirm a very poor morphological integration of the cranial-cervical-maxillary complex in individuals with DS. This finding reinforces the proposal that gene overload enhances the channeling process.

## 1. Introduction

Down syndrome (DS) is a disorder characterized by the presence of an additional chromosome or a part of the same in position 21, which causes a characteristic phenotype, intellectual disability and several physical and medical peculiarities [1]. The orofacial manifestations of DS are characteristic of the syndrome and can affect soft and hard tissues, jeopardizing eating, chewing, swallowing, and phonation [2]. One of the inherent characteristics of the aneuploid condition is the high phenotypic variability, which has been described in relation to the cognitive capacity, hypotonia, dermatoglyphic findings, susceptibility to certain comorbidities and facial dysmorphology [3]. The phenotypic variability can be structured according to three interrelated components: developmental stability, morphological integration, and canalization [4].

Stability is a property of development that can limit phenotypic variation when faced with random perturbations (“developmental noise”) that occur during the course of ontogeny, such that a certain phenotype is produced under specific genetic and environmental conditions [5,6]. When the genetic or environmental stress increases the developmental noise, minor deviations occur in the ideal developmental program of perfect bilateral symmetry, and developmental instability occurs [4]. Historically, developmental instability has been evaluated using an analysis of fluctuating asymmetry, defined as a measure of the metric differences between the left and right sides of developing organisms [7,8].

Modularity is an important organizational component in biological systems that describes the differences in the degree of trait integration between and within groups and subgroups of structures and that is manifested at the morphological level [9]. Morphological integration refers to the tendency of various traits to show correlated variations because they develop in response to shared development processes or work in concert with other shared structures [10,11]. This concept was introduced by Olson and Miller [10] who argued that those traits that share developmental aspects (tissue precursors, development chronology, topological proximity in the embryo, etc.) or similar functional demands (chewing, vision, locomotion, etc.) will evolve as integrated units.

In the 1940s, Conrad H. Waddington [12] employed the term “canalization” to describe a phenotype’s ability to resist perturbations. Canalization implies that a genotype’s phenotype remains relatively unchanged when the individuals of a particular genotype are exposed to various environments (environmental canalization) or when the individuals of the same genotype differ in their genetic origin (genetic canalization). Developmental instability and canalization perform a relevant role in micro- and macro-evolutionary processes [8]. Developmental instability is expressed phenotypically by the intraindividual variation, while canalization is expressed by the interindividual variation. The combined effect of developmental instability and canalization has been called developmental homeostasis [13]. It has been suggested that they share a common background but should be considered distinct processes until their origins and underlying mechanisms have been definitely clarified [13].

The deficient development that characterizes DS also affects the head, which is generally small and has a tendency toward a brachycephalic pattern [14]. The cranial base is flattened, and its length is shorter than that of the general population [15]. Between 10% and 30% of individuals with DS have radiological findings suggestive of atlantoaxial instability, although most cases are asymptomatic [16]. Hypotonia and ligamentous laxity in DS can lead to a substantial fluctuation in the craniovertebral junction, not only between the atlas and axis but also between the occipital and atlas. It has therefore been suggested that it would be more correct to use the term craniovertebral instability [17]. The maxilla is typically hypoplastic [18], which translates into a deficient development of the middle third of the face [19]. A reduced size has also been reported both for the ramus [18] and mandibular body [15,18]. It has been suggested that there is authentic cranial dysplasia in DS, which results in a high prevalence of class III skeletal malocclusions, becoming more apparent with age [19,20]. The cranium presents modular organization, consisting of distinct and semi-independent functional units, which interact substantially during ontogeny, consistent with the principles of morphological integration [21,22,23,24,25]. In DS, however, several authors considered that the overexpression of certain genes encoded in chromosome 21 boost canalization [26], while others argue that trisomy 21 causes amplified developmental instability [27]. In short, the participation and, if applicable, prominence of these mechanisms in the integration of the cranial-vertebral-maxillary complex in individuals with DS is still controversial.

This study aimed to analyze whether there are significant relationships between the skull base, atlantoaxial joint and maxillary-mandibular complex in a group of individuals with DS, assuming that these structures behave as modules, given that they share developmental precursors, are located spatially close to each other, participate jointly in various functions and have a common evolutionary history. The null hypothesis is that there is no significant morphological integration between the skull base, atlantoaxial joint and maxillary-mandibular complex.

## 2. Materials and Methods

### 2.1. Participant Selection

The study group of convenience consisted of 41 individuals with DS (15 female and 26 male participants) between the ages of 9 and 43 years. The mean age of the female participants was 17.2 ± 5.3 years (range 7–29 years) and that of the male participants was 19.9 ± 8.3 years (range 9–43 years). The mean age of the female participants was 17.2 ± 5.3 years (range 7–29 years) and that of the male participants was 19.9 ± 8.3 years (range 9–43 years). The age distribution of the study group was as follows: 9–15 years, 12 individuals; 16–20 years, 19 individuals; 21–25 years, 4 individuals; 26–30 years, 2 individuals; 31–35 years, 2 individuals; and 36–40 years, 2 individuals. All participants had undergone cone-beam computed tomography (CBCT) within the framework of a previous study conducted in the Faculty of Medicine and Odontology of the University of Santiago de Compostela (Spain).

The inclusion criteria were as follows: not having undergone previous orthopedic/orthodontic treatment, not having undergone maxillofacial surgery involving the maxillary bones and having images of the cranial-cervical-facial complex of acceptable quality in the three orthogonal spatial planes. These images should allow for a complete cephalometric tracing that includes the following end points: (1) in the anteroposterior direction, from the anterior nasal spine to the most caudal portion of the occipital lamina, and (2) in the vertical axis, from the basion to at least the base of the axis.

Applying the same inclusion criteria, we selected a control group of 41 individuals without systemic disease and without severe maxillofacial bone malformations, paired by sex (15 female and 26 male patients) and age (the mean age was 16.5 ± 5.7 years (range 8–29 years) for the female participants and 18.8 ± 8.5 years (range 9–43 years) for the male participants) with the study group.

The participants (or their legal guardians, if necessary) signed an informed consent authorizing the use of the images. The study design was approved by the Ethics Committee of the University of Santiago de Compostela.

### 2.2. Radiological Measurements

The images were obtained using an i-CAT^®^ cone-beam computed tomography system (Imaging Sciences International, Hatfield, PA, USA). The imaging studies were performed by a single operator, following the manufacturer’s instructions, and standardizing the patients’ position. To perform the scanning, the participants were placed in the standing position, with the intermaxillary occlusion in maximum intercuspation and with the following head position: in the sagittal plane, with the Frankfurt plane parallel to the floor (anterior and posterior nasal spine aligned and coinciding with the horizontal), and in the frontal plane, with the vertebral body aligned with the vertical axis.

The images were obtained at 120 kVp and 5.0 mA; the voxel size was 0.3 mm, with a field of view of 10–20 cm and an exposure time of 8.9 s. All images were reconstructed using i-CAT^®^ Vision 17.1 software (Imaging Sciences International, Hatfield, PA, USA) and were exported in DICOM format (Digital Imaging Communication in Medicine) to a personal computer with MacBook 27 software (MacOS X 10.6, Apple Inc., Cupertino, CA, USA). All measurements were performed using the open-source, image-processing software OsiriX (Pixmeo, Geneva, Switzerland).

The CBCT scans were oriented by aligning the palatal vault with the X axis (in the sagittal and coronal planes) and the midpalatal suture with the Y axis (in the axial and coronal planes). The cursor was then moved within the plane that was the object of the study following the Z axis (the selected coronal plane was that which passed through the mesial face of the first maxillary molars in the axial perspective). The images were analyzed in the sagittal and frontal planes: the sagittal plane was that which crossed the midpoint of the odontoid apophysis and the facial midline; the frontal plane was established based on the highest point of the odontoid apophysis.

Twenty-nine measurements grouped into three blocks were performed on each patient (Table S1). The first block of atlantoaxial (A) dimensions (9 measurements) were as follows: sagittal atlantodens interval, left mesial atlantoaxial interval, left medial atlantoaxial interval, left lateral atlantoaxial interval, right mesial atlantoaxial interval, right medial atlantoaxial interval, right lateral atlantoaxial interval, left lateral atlantodens interval and right lateral atlantodens interval. The second block of craniocervical dimensions (B) (7 measurements) were as follows: Wackenheim measurement, McRae measurement, Chamberlain measurement, McGregor measurement, Redlund-Johnell method, modified Ranawat method and length of the odontoid apophysis. The third block of cephalometric dimensions (C) (13 measurements) were as follows: McRae–Wackenheim angle; length, anterosuperior and vertical position, and inclination of the maxilla; angle between the axis of the maxillary central incisor and the palatal plane; size, anterosuperior and vertical position, and inclination of the mandible; angle between the axis of the mandibular central incisor and the Downs mandibular plane; and the distance between points A and B projected on the McRae line.

To check the intraexaminer repeatability, a single observer repeated the measurements of 10 randomly selected cases (5 patients with DS and 5 controls) one month after the first measurements, obtaining an intraclass correlation coefficient of 0.94 (95% CI 0.84–0.96) for all evaluated variables. To analyze the interobserver reproducibility, two observers performed all the measurements in 10 other randomly selected cases (5 patients with DS and 5 controls), obtaining an intraclass correlation coefficient of 0.92 (95% CI 0.72–0.99). The lowest values corresponded to variables AA1-L and AA1-R (0.75 and 0.72, respectively).

### 2.3. Statistical Methodology

We conducted an analysis of the differences between the cases and controls to determine whether it was possible to discriminate between the two groups based on the values of the variables for each of the three examined blocks. Basically, we sought to find a linear combination of response variables that maximized the differences between the cases and controls. To this end, we performed a multivariate analysis of variance to assess the presence of intergroup differences in the means and then performed a linear discriminant analysis to obtain the linear combination of variables that maximizes the intergroup discrimination.

To analyze the association of the measurements between pairs of blocks of variables (A vs. B, A vs. C, B vs. C), we selected the canonical correlation analysis methodology because this method allows for the sequential extraction of linear combinations of variables (measured in the same set of individuals) searching for the maximum sample correlation. The objective of the analysis is to obtain the canonical dimensions (the number of dimensions equals the number of variables in the smaller block) and to test for their significance. We used the Pillai–Barlett Trace using the F-approximation to reach this goal. The estimation of the numerator and denominator degrees of freedom of the test takes into account the sample size and the dimension of the blocks (number of variables in each block). The canonical correlation between the sets of items of each dimension was also estimated.

## 3. Results

The measurements of centralization and dispersion of the atlantoaxial, craniovertebral and cephalometric dimensions in the DS and control groups are detailed in Table 1, Table 2 and Table 3 respectively.

The atlantoaxial dimensions of the DS group were significantly smaller than those of the control group (*p* < 0.001). The craniovertebral dimensions of the DS group were significantly larger than those of the control group (*p* < 0.001), except for the length of the odontoid apophysis, which was significantly shorter in the DS group (*p* < 0.001). The cephalometric dimensions of the DS group differed significantly from those of the control group (*p* < 0.001).

The capacity to discriminate whether an individual belonged to one or the other group was 64% based on the atlantoaxial dimensions, 83% based on the craniovertebral dimensions and 87.5% based on the cephalometric dimensions.

Table 4 and Table 5 show the results of the dimensionality test for each pair of blocks in the DS and control groups. The first test (dimension 1) represents the tests between the six dimensions combined, the second test from two to six, and so on, stopping when the sixth dimension alone is tested. The significant canonical dimensions explain the relationship between the variables in the pair of blocks.

When relating (by pairs) the various measurement blocks (A, atlantoaxial; B, craniovertebral; C, cephalometric), the dimensionality test for the canonical analysis of the DS group shows that none of the six canonical dimensions was statistically significant at the 0.05 level in any of the pairs (Table 4), which implies that the measurement blocks in the DS group were independent of each other. In the control group by contrast, 1 (group A vs. B), 4 (group A vs. C) and 5 (group B vs. C) canonical dimensions were statistically significant, with the higher correlations between blocks A and B related to C (Table 5), which implies that the measurement blocks performed in the nonsyndromic individuals are interdependent.

## 4. Discussion

This study showed that the atlantoaxial, craniovertebral, and cephalometric dimensions of the individuals with DS differed significantly from those recorded in the nonsyndromic individuals. In contrast to the control group, the three measurement blocks in the DS group were independent of each other.

The hypothesis has been suggested that trisomy 21 causes “amplified developmental instability”, which results in increased asymmetry and variability of the phenotypic characteristics [28,29]. In DS, an increase in fluctuating asymmetry has been reported in skeletal abnormalities [30], dermatoglyphic abnormalities [28], palatal dimensions [31] and facial dysmorphology [27].

Facial morphogenesis requires adequate spatiotemporal deployment of gene products, neural crest cells, and other cells to develop the facial prominences [32]. These prominences have to be established, grow and fuse in a coordinated manner to form the structures that will comprise a functional craniofacial complex [33]. Trisomy causes a generalized genetic imbalance that interrupts the evolutionarily preserved morphogenetic pathways of development [34]. This mechanism would explain the onset of orofacial abnormalities, by formation, growth, and/or fusion defects of the facial prominences during embryonal craniofacial development [34]. Research conducted on Ts65Dn mice, which are trisomic for the orthologs of approximately half of the genes found in the human chromosome 21 and exhibit craniofacial abnormalities similar to DS, confirmed that trisomy 21 was a neurocristopathy that involves cells that give rise to facial prominences [35].

Starbuck et al. [16] reported that facial prominences in DS showed an increase in fluctuating asymmetry during facial morphogenesis, which provides arguments for an increase in facial developmental instability in patients with DS. According to these authors [16], the imbalance caused by trisomy variably affects the facial regions derived from the embryonic facial prominences, therefore jeopardizing the development of these regions rather than causing a generalized interruption in development, as has been previously suggested [36].

The proponents of the hypothesis of amplified developmental instability [28,37,38] hold that the abnormal phenotypes found in individuals with DS (such as smaller palates and dental abnormalities) tend to be the same as those that are less damped (or canalized) during the developmental period in euploid individuals. Therefore, these especially labile traits are more likely to exhibit increased phenotypic variation when they are subjected to genetic or environmental aggressions during development [28,39].

However, Starbuck et al. [40] demonstrated that the faces of children with DS were quantitatively more similar to those of their siblings than to those of unrelated euploid individuals, and the majority showed variations in the normal variation range established among euploid samples. One of this study’s conclusions, as well as revealing the genetic foundations of the similarity between relatives, was to confirm the resistance of craniofacial development to genetic perturbations caused by trisomy 21 [40]. After performing a rigorous biostatistical analysis of the dental dimensions in individuals with DS, a study published by Matabuena Rodríguez et al. [41] concluded not only that fluctuating dental asymmetry was less in DS than in the general population but also that several dental morphometric variables could also be more stable in individuals with trisomy 21. These findings allow us to speculate that canalization in DS could prevail over fluctuating asymmetry.

Another study published by Starbuck et al. [42], whose objective was to study the effects of trisomy 21 on the covariance patterns of facial measures, compared the morphological integration patterns of immature faces of patients with DS and those of their nonsyndromic siblings. The association patterns of linear distances in the upper and middle portions of the face do not seem to be affected by trisomy 21; however, the association patterns of linear distances in the lower part of the face were significantly different between the two groups of individuals. Extrapolating these findings from soft tissues to hard tissues, one could explain why in the present study the values of the cephalometric measurements (which mainly affect the lower third of the face) are those with greater capacity to discriminate whether an individual has DS or belongs to the control group.

Starbuck et al. [42] also found statistically significant differences in terms of morphological integration between various facial regions in the patients with DS, unlike their nonsyndromic siblings. This finding coincides to a degree with our results, given that we found no significant relationship between the cranial-cervical-maxillary measurement blocks in the DS group but did find them in the paired controls.

Pritchard and Kola [26] defended the “gene dosage effect” hypothesis to explain the DS phenotype, to the detriment of the “amplified developmental instability” hypothesis. According to these authors, the phenotypic traits of the aneuploid syndromes and of DS in particular are the result of an overexpression of specific genes encrypted in chromosome 21. Our results confirm that cranial-cervical-maxillary morphological integration is very poor in the individuals with DS, which allows us to speculate that genetic overload boosts the canalization process, therefore granting certain autonomy to the functional units during the growth period.

This study is not exempt from several limitations that should be considered when interpreting the results. All of the participants underwent a CBCT examination, which could entail a selection bias based on their degree of cooperation [31], given that the patients with more severe phenotypes, who presumably have an increased degree of intellectual disability and consequently are less cooperative, could have been involuntarily excluded. CBCT is an indispensable tool for obtaining dental-maxillary-facial images [43]; however, very few studies have been published with this technique to compare with our results. Although CBCT is not justified in any case for exclusively research procedures [44], the images in our series belonged to the historic file of the University of Santiago de Compostela. The participants likely had to wear a cervical collar while the radiological scan was being performed to standardize the neck position, as several authors have suggested [45].

## 5. Conclusions

The atlantoaxial, craniovertebral, and cephalometric dimensions of individuals with DS differ significantly from those of nonsyndromic controls. Unlike the general population, the individuals with DS had no statistically significant relationship between these three blocks of dimensions. This confirms that in DS, the cranial-cervical-maxillary morphological integration is very poor, reinforcing the hypothesis of genetic overload to explain the DS phenotype. Additionally, these findings could affect the decision making and the long-term results of certain interventions in this anatomical region, such as maxillofacial orthopedics and orthognathic surgery.

## Figures and Tables

**Table 1:** Atlantoaxial Measurements in the Study (Down Syndrome) and Control Group.

Measurement *	Mean	SD	Median	Minimum	Maximum
**Study Group (Down Syndrome)**					
**ADI**	0.189	0.097	0.149	0.056	0.409
**AA1. L**	0.165	0.077	0.164	0.034	0.346
**AA2. L**	0.203	0.088	0.208	0.035	0.371
**AA3. L**	0.151	0.065	0.143	0.054	0.318
**AA1. R**	0.186	0.074	0.180	0.074	0.361
**AA2. R**	0.224	0.073	0.225	0.113	0.469
**AA3. R**	0.153	0.070	0.143	0.059	0.345
**ADL. L**	0.431	0.135	0.417	0.153	0.719
**ADL. R**	0.439	0.176	0.435	0.140	1.020
Control Group					
**ADI**	0.181	0.052	0.167	0.120	0.412
**AA1.L**	0.209	0.068	0.186	0.091	0.381
**AA2.L**	0.262	0.055	0.262	0.184	0.415
**AA3.L**	0.196	0.058	0.206	0.104	0.338
**AA1.R**	0.206	0.094	0.177	0.056	0.474
**AA2.R**	0.262	0.078	0.250	0.149	0.585
**AA3.R**	0.190	0.075	0.185	0.115	0.458
**ADL.L**	0.567	0.847	0.368	0.245	4.190
**ADL.R**	0.565	0.674	0.420	0.293	3.440

* The linear measurements are expressed in centimeters. SD, standard deviation; ADI, atlantodens interval; AA1.L, left mesial atlantoaxial interval; AA2.L, left medial atlantoaxial interval; AA3.L, left distal atlantoaxial interval; AA1.R, right mesial atlantoaxial interval; AA2.R, right medial atlantoaxial interval; AA3.R, right distal atlantoaxial interval; ADL.L, left lateral atlanto-dens interval; ADL.R, right lateral atlanto-dens interval.

**Table 2:** Craniovertebral Measurements in the Study (Down Syndrome) and Control Group.

Measurement *	Mean	SD	Median	Minimum	Maximum
Study Group (Down Syndrome)					
**Wackenheim**	0.212	0.207	0.198	−0.170	0.831
**McRae**	0.503	0.174	0.529	0.162	0.837
**Chamberlain**	0.332	0.307	0.349	−0.280	1.000
**McGregor**	0.255	0.297	0.270	−0.390	0.929
**Redlund-Johnell**	3.589	0.442	3.556	2.670	4.436
**Ranawat**	2.696	0.306	2.741	2.000	3.459
**L. odontoids**	3.358	0.346	3.385	2.742	4.000
Control Group					
**Wackenheim**	0.142	0.152	0.140	−0.160	0.375
**McRae**	0.263	0.148	0.200	0.107	0.642
**Chamberlain**	0.042	0.350	0.121	−0.420	0.762
**McGregor**	−0.060	0.372	0.047	−0.580	0.653
**Redlund-Johnell**	3.430	0.261	3.412	2.950	4.020
**Ranawat**	2.854	0.232	3.000	2.190	3.202
**L. odontoids**	3.577	0.274	3.520	2.950	3.930

* The linear measurements are expressed in centimeters. SD, standard deviation, Wackenheim, Wackenheim measurement; McRae, McRae measurement; Chamberlain, Chamberlain measurement; McGregor, McGregor measurement; Redlund-Johnell, Redlund-Johnell method; Ranawat, modified Ranawat method; L. odontoids: length of the odontoid apophysis.

**Table 3:** Cephalometric Measurements in the Study (Down Syndrome) and Control Group.

Measurement *	Mean	SD	Median	Minimum	Maximum
**Study Group (Down Syndrome)**					
**McRae-Wac**	54.089	7.437	53.468	40.890	71.767
**ENA-ENP**	4.285	0.501	4.202	3.420	5.540
**A-P-McRae**	8.644	0.600	8.456	7.590	9.880
**A-McRae**	1.001	1.111	0.775	−0.580	5.160
**McRae-PP**	5.382	3.732	4.722	0.000	14.780
**ICS-PP Axis**	69.069	15.578	66.455	51.100	119.000
**Co-Go**	5.375	0.654	5.465	3.818	6.700
**Go-Po**	6.609	0.575	6.620	5.470	7.720
**B-P-McRae**	8.626	0.844	8.500	7.390	10.600
**McR-BaGn**	36.959	6.828	35.770	25.981	53.500
**Branch-Body**	119.766	7.709	120.564	102.569	134.561
**II-GoMe Axis**	89.670	22.027	94.169	−8.582	111.580
**A-B**	0.293	0.463	0.230	−0.852	1.750
Control Group					
**McRae-Wac**	60.403	5.194	60.590	53.472	73.819
**ENA-ENP**	5.110	0.588	5.205	3.673	5.820
**A-P-McRae**	8.334	1.026	8.401	6.980	10.180
**A-McRae**	0.915	1.005	0.796	−1.093	2.160
**McRae-PP**	4.799	4.683	3.230	1.570	20.265
**ICS-PP Axis**	68.245	8.592	68.230	56.160	85.247
**Co-Go**	5.516	0.492	5.531	4.790	6.300
**Go-Po**	7.615	0.926	7.314	5.770	8.880
**B-P-McRae**	8.336	0.802	7.896	7.500	9.860
**McR-BaGn**	37.775	4.776	39.270	22.441	44.980
**Branch-Body**	126.610	11.697	126.808	108.680	140.580
**II-GoMe Axis**	84.509	12.898	81.460	64.740	104.800
**A-B**	0.713	0.291	0.844	0.178	1.190

* The linear measurements are expressed in centimeters, and the angular measurements are expressed in degrees. SD, standard deviation; McRae-Wac, McRae–Wackenheim angle; ENA-ENP, palatal plane; A-P-McRae, A-perpendicular to McRae; A-McRae, distance between A-McRae; McRae-PP, McRae-Palatal Plane; ICS-PP Axis, ICS-palatal plane axis; Co-Go, Condilion-Gonion distance; Go-Po, Gonion-Pogonion distance; B-P-McRae, B-perpendicular to McRae distance; McR-BaG, angle between McRae and Basion-Gnathion line; Branch-Body, angle between mandibular branch tangent-mandibular body tangent; II-GoMe Axis, angle between the axis of the mandibular central incisor and the Downs mandibular plane (Gonion-Menton); A-B, distance between points A and B.

**Table 4:** Results of the Canonical Correlation Analysis when Comparing by Pairs the Blocks of Measurements Performed in the Down syndrome Group.

Block A *versus* Block B						
Dimension	Can. Corr.	Pillai’s trace	F approx	DF1	DF2	*p* value
1	0.831	1.282	1.087	36	144	0.356
2	0.550	0.590	0.681	25	156	0.870
3	0.438	0.288	0.529	16	168	0.929
4	0.273	0.096	0.324	9	180	0.966
5	0.137	0.021	0.171	4	192	0.953
6	0.051	0.003	0.088	1	204	0.766
**Block A *versus* Block C**						
Dimension	Can. Corr.	Pillai’s trace	F approx	DF1	DF2	*p* value
1	0.665	1.205	0.838	36	120	0.725
2	0.634	0.763	0.769	25	132	0.774
3	0.436	0.361	0.575	16	144	0.898
4	0.374	0.170	0.507	9	156	0.868
5	0.175	0.031	0.217	4	168	0.929
6	0.011	0.000	0.003	1	180	0.953
**Block B *versus* Block C**						
Dimension	Can. Corr.	Pillai’s trace	F approx	DF1	DF2	*p* value
1	0.905	1.765	1.319	36	114	0.137
2	0.601	0.945	0.942	25	126	0.548
3	0.562	0.583	0.929	16	138	0.538
4	0.445	0.268	0.779	9	150	0.636
5	0.260	0.070	0.475	4	162	0.754
6	0.045	0.002	0.060	1	174	0.807

Block A, atlantoaxial measurements; Block B, craniovertebral measurements; Block C, cephalometric measurements; Can. Corr, Canonical Correlation; DF1, degrees of freedom (numerator); DF2, degree of freedom (denominator); F approx., approximate F statistic.

**Table 5:** Results of the Canonical Correlation Analysis when Comparing by Pairs the Blocks of Measurements Performed in the Control Group.

Block A *versus* Block B						
Dimension	Can. Corr.	Pillai’s trace	F approx	DF1	DF2	*p* value
1	0.998	2.467	1.746	36	90	0.018
2	0.896	1.471	1.325	25	102	0.165
3	0.669	0.667	0.892	16	114	0.580
4	0.379	0.220	0.534	9	126	0.848
5	0.271	0.077	0.448	4	138	0.774
6	0.059	0.003	0.086	1	150	0.769
**Block A *versus* Block C**						
Dimension	Can. Corr.	Pillai’s trace	F approx	DF1	DF2	*p* value
1	0.990	3.985	3.956	36	72	<0.001
2	0.990	2.985	3.327	25	84	<0.001
3	0.990	1.985	2.967	16	96	<0.001
4	0.875	0.985	2.358	9	108	0.017
5	0.461	0.220	1.140	4	120	0.340
6	0.086	0.007	0.162	1	132	0.680
**Block B *versus* Block C**						
Dimension	Can. Corr.	Pillai’s trace	F approx	df1	df2	*p* value
1	0.990	4.560	5.806	36	66	<0.001
2	0.990	3.560	4.552	25	78	<0.001
3	0.990	2.560	4.186	16	90	<0.001
4	0.990	1.560	3.982	9	102	<0.001
5	0.740	0.560	2.934	4	114	0.020
6	0.110	0.012	0.254	1	126	0.610

Block A, atlantoaxial measurements; Block B, craniovertebral measurements; Block C, cephalometric measurements; Can. Corr., Canonical Correlation; DF1, degrees of freedom (numerator); DF2, degrees of freedom (denominator); F approx., approximate F statistic.

## Data Availability

The data that support the findings of this study are available on request from the corresponding authors.

## Supplementary Materials

The following supporting information can be downloaded at: https://www.mdpi.com/article/10.3390/biology11040496/s1, Table S1: Atlantoaxial, Craniocervical and Cephalometric Measurements Performed on cone-beam computed tomography images [45,46,47,48,49,50,51,52,53,54,55,56,57,58,59,60,61,62,63,64,65,66,67,68].
