# Supplementary material for: Cranial-Vertebral-Maxillary Morphological Integration in Down Syndrome
Source: Biology (Basel). 2022 Mar 24;11(4):496. doi: 10.3390/biology11040496 (PMC9027221; doi:10.3390/biology11040496)
Supplement: Supplementary file 1 [file biology-11-00496-s001.zip › biology-1585388-supplementary.pdf]

Table S1. Atlantoaxial, Craniocervical and Cephalometric Measurements Performed on cone beam computed tomography images

**ATLANTOAXIAL DIMENSIONS**	
**Sagittal Plane**<br>• *Sagittal Atlanto-Dens Interval (yellow line):* This is the distance from the midpoint of the joint surface of the atlas to the closest zone of the adjacent area of the apophysis odontoid [46].	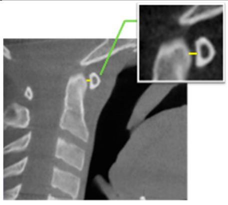
**Frontal Plane**<br>• *Atlantoaxial Interval (yellow solid lines):* This is the distance between the upper surface of the body of the axis and the base of the lateral masses of the atlas at the mesial, medial and lateral levels, both on the right and left sides [47].<br>• *Lateral Atlanto-Dens Interval (yellow dotted lines).* This was measured on both the right and left sides and is the distance between the internal surface of the lateral masses of the atlas and the odontoid apophysis in the midpoint of the body of the vertebra [45].	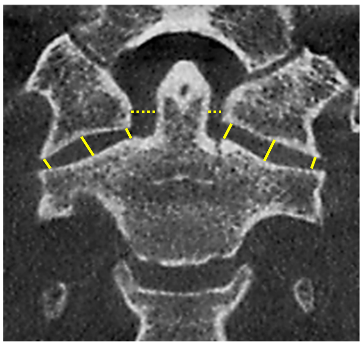
**CRANIOCERVICAL DIMENSIONS**	
**Sagittal Plane**<br>• *Wackenheim measurement (yellow line):* the minimum distance between the odontoid apophysis and the Wackenheim line. This line passes through the caudal extension of the dorsal surface of the clivus (green line)[48].	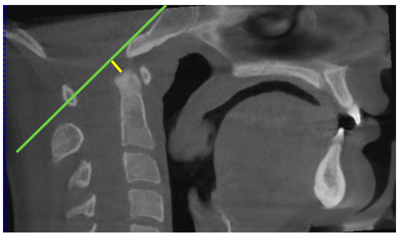
• *McRae measurement (yellow line):* the perpendicular distance between the end of the odontoid apophysis and the McRae line. The McRae line or foramen magnum line joins the anterior (basion) and posterior margins (opisthion) of the foramen magnum (green line) [49].	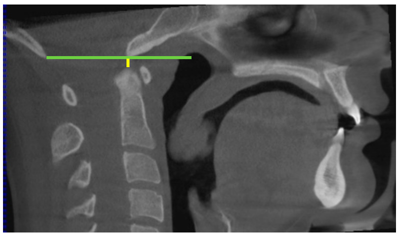

• *Chamberlain measurement (yellow line):* the perpendicular distance between the end of the odontoid apophysis and the Chamberlain line. This line extends from the posterior nasal spine to the posterior margin of the foramen magnum (opisthion) (green line) [50].	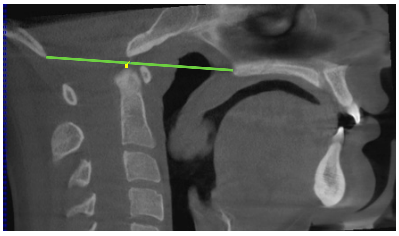
• *McGregor measurement (yellow line):* the perpendicular distance between the end of the odontoid apophysis and the McGregor line. This line joins the posterior nasal spine with the most caudal point of the squamous surface of the occipital bone (green line) [51].	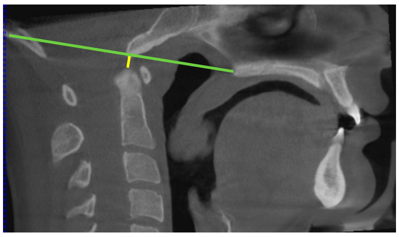
• *Redlund-Johnell method (yellow line):* the minimum distance between the midpoint of the base of the axis (C2) and the McGregor line (green line) [52].	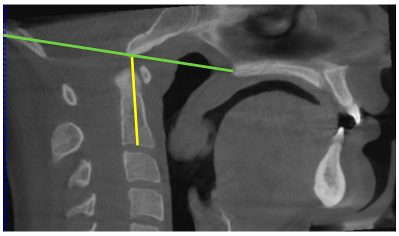
• *Modified Ranawat method (yellow line):* Along the entire longitudinal axis (C2), this method measures the distance between the midpoint of the base of C2 and a line that runs between the center of the anterior arch of the atlas (C1) and the center of the posterior arch of the atlas (green line) [53].	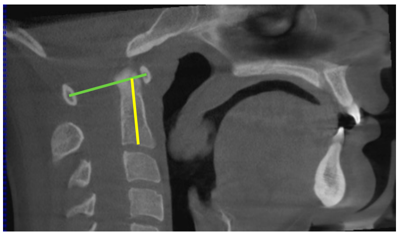
• *Length of the odontoid apophysis (yellow line):* the distance between the midpoint of the base of C2 and the end of the odontoid apophysis [54].	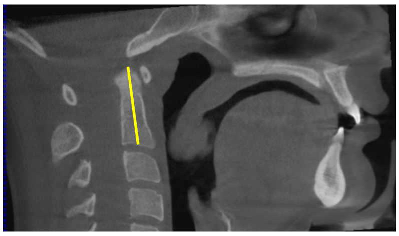

CEPHALOMETRIC DIMENSIONS	
• *McRae-Wackenheim angle (yellow curved line):* formed by the McRae and Wackenheim lines (green lines) and expressed in degrees [55].	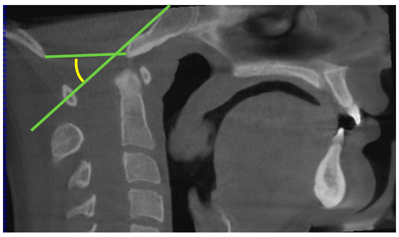
• *Palatal plane (yellow line):* provides information on the posterior-anterior length of the maxilla. This is the distance between the anterior and posterior nasal spines [56,57].	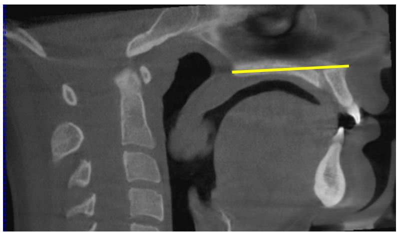
• *A-perpendicular to McRae (yellow line):* provides information on the posterior-anterior position of the maxilla. This is the distance from point A of the maxilla to the perpendicular (green dotted line) of the McRae line that passes through the basion (green solid line). Point A is the deepest part of the anterior notch of the maxilla in the sagittal plane (white dot) [58,59].	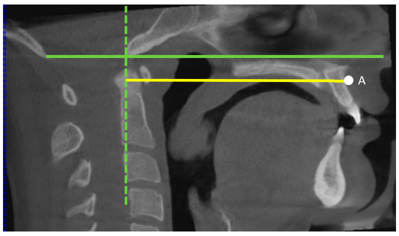
• *A--McRae distance (yellow line):* provides information on the vertical position of the maxilla. This is the distance between point A (white dot) and the McRae line (green line) [58].	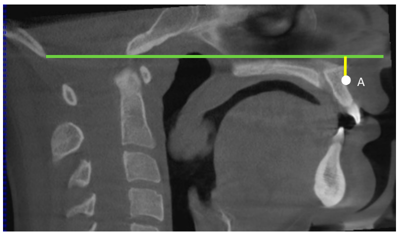
• *McRae-Palatal plane (yellow curved line):* provides information on the inclination of the maxilla. This is the angle between the McRae line (green dotted line) and the palatal plane, translated to the Basion point (green solid line) [60,61].	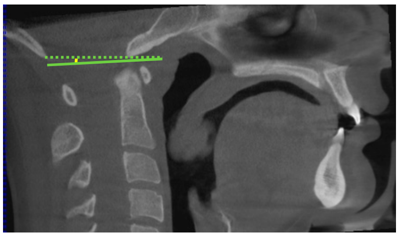

• *ICS-Palatal plane axis (yellow curved line):* the angle between the axis of the maxillary central incisor and the palatal plane [58].	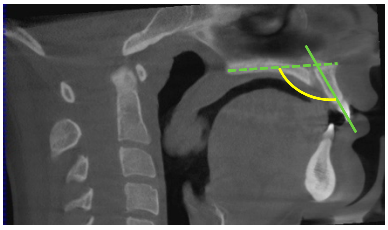
• *Length of the mandibular ramus (Co-Go) (yellow line):* provides information on the size of the mandible. This is the distance between the Condilion (Co) and Gonion points (Go) [62–64]. The Co point is the most posterior-superior point of the mandibular condyle.	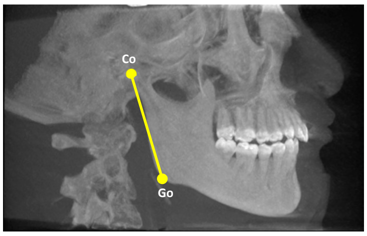
• *Length of the mandibular body (Go-Pg) (yellow line):* provides information on the size of the mandible. This is the distance between the Gonion (Go) and Pogonion points (Pg) [62–64]. The Go point is the most posterior-inferior point of the mandibular gonial angle. The Pg point is the most anterior point of the mandibular symphysis in the sagittal plane.	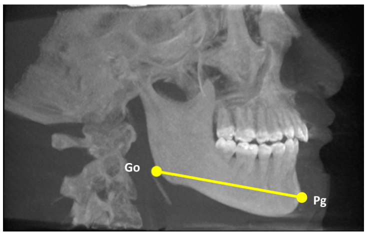
• *B-perpendicular to McRae (yellow line):* provides information on the posterior-anterior mandibular position. This is the distance from point B of the mandibular symphysis to the perpendicular (green dotted line) of the McRae line that passes through the Basion point (green solid line) [58]. Point B is the deepest point of the mandibular symphysis in the sagittal plane.	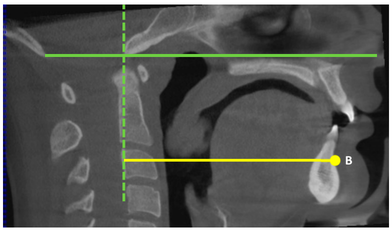

• *McRae-BaGn (yellow line):* provides information on the vertical mandibular position. This is the angle formed by the McRae (green solid line) and Basion-Gnathion line (green dotted line) [65–67]. The Gnathion is the most anterior-inferior point of the edge of the mandibular symphysis in the sagittal plane.	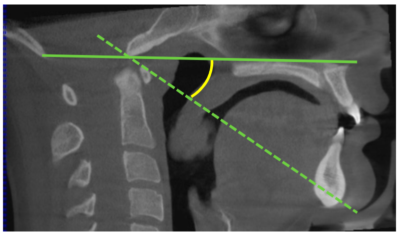
• *Mandibular ramus tangent-mandibular body tangent (yellow curved line):* provides information on mandibular growth. This is the angle formed by the tangent to the posterior edge of the mandibular ramus (green solid line) and by the tangent to the inferior edge of the mandibular body that passes through the Menton (green dotted line), which is the lowest point of the mandibular symphysis in the sagittal plane [62–64].	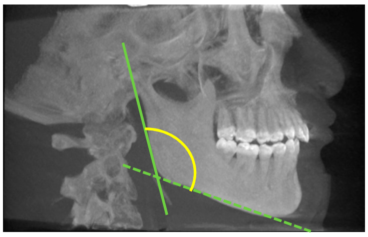
• *The ICI-GoMe axis (yellow curved line):* the angle formed by the axis of the mandibular central incisor (green solid line) and the Downs mandibular plane (Gonion-Menton) (green dotted line) [68].	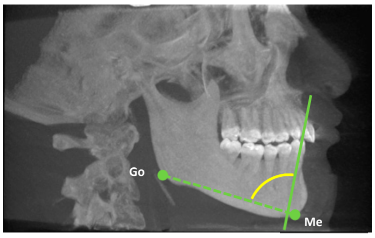
• *A-B distance (yellow line):* To analyze the maxillary-mandibular relationship, we determined the distance between points A (maxillary) and B (mandibular) projected on the McRae line (green dotted line) [58].	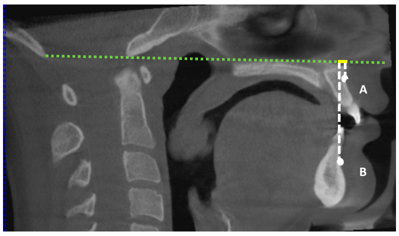
